# Society Meetings

**Published:** 1913-07

**Authors:** 


### SOCIETY MEETINGS.

*Secretaries of County and other medical societies of western New York—Buffalo to Utica—are requested to send list of officers, and general information as to dates and places of meetings, so that they may be published in the August and September issues.*

---

### The Campaign Against Cancer

A National Anti-Cancer Association was organized at a meeting of physicians and laymen at the Harvard Club, Boston, May 22. Various national societies of specialists were represented. The following officers were elected: President, George C. Clark, New York; vice-Presidents, Dr. Clement Cleveland, New York; Dr. L. M. McMurtry, Louisville; Dr. Edward Reynolds, Boston; Dr. Edward Martin, Philadelphia; Dr. L. F. Barker, Baltimore; Secretary, T. M. Debevoise. The chairman of the Executive Committee is Dr. G. E. Brewer, New York, and other members of the committee are Drs. J. E. Simpson, Pittsburgh; Joseph Bloodgood, Baltimore; A. D. Bevan, Chicago; Jeff Miller, New Orleans; Charles Power, Denver; F. J. Taussig, St. Louis, and Reuben Peterson, Ann Arbor. There is also a laymen's committee, headed by James Speyer of New York.

Without wishing to place an obstacle in the way of any movement toward the prevention of disease, it ought to be clearly recognized that the time is not ripe to attack this problem from the standpoint of etiology except as laymen can be impressed with the importance of assisting research and of realizing the frank agnosticism of most of the profession in this regard and the fact that the profession is not to blame for its ignorance any more than any other science is to blame for having unsolved problems and limitations of knowledge. Neither is there any method of treatment generally recognized as efficient except operation and certain radiant and caustic methods of limited applicability. Even under the best circumstances the practical results of treatment are less satisfactory than for many other diseases, even those till recently considered of bad prognosis. But the Association is needed to disseminate information as to the danger of neglecting early symptoms and of trusting to inefficient means of treatment by incompetent quacks and, along these lines, many valuable lives can be saved and much unnecessary suffering avoided.

---

Medical Society of Erie County. The regular meeting was held June 16. After routine business the following papers were read: "Treatment of Breech Presentation," Dr. P. W. Van-Peyma; "Forests and Their Relationships to Eugenics," Dr. George N. Jack.

---

The Buffalo Academy of Medicine has elected the following officers for the year 1913-14:

Section of Medicine—Chairman, Dr. Herman K. De Groat; Secretary, Theodore M. Leonard.

Section of Surgery—Chairman, W. W. Plummer; Secretary, Dr. Charles W. Bethune.

Section of Gynaecology and Obstetrics—Chairman, Descum C. McKenney; Secretary, John A. Ragone.

Section of Pathology, Etc.—Chairman, A. A. Thibaudeau; Secretary, Bernard F. Schreiner.

General Academy Officers—President, Harry R. Trick; vice-Presidents, ex-officio, the Chairman of Sections; Secretary, Herbert A. Smith; Treasurer, Lawrence Hendee; Trustee, Thomas F. Duyer.

---

The Fourth International Congress on School Hygiene will meet in Buffalo Aug. 25-30. Two hundred and thirty papers and ten symposiums are promised. Let us trust that the curricula for school children will not be arranged on the same liberal basis. This plethora will doubtless be depleted before the final program is arranged. The medical profession is cordially invited to attend and to take a permanent interest in the organizations represented.

---

The Elmira Academy of Medicine at its meeting of June 4 enjoyed the following program: "Indicated Remedies—Ecchinacea, Cactus and Gelsemium," M. H. Potts, M. D.; "A Brief Resume of Medical Progress During the Past Twenty-five Years," La Rue Colgrove, M. D.; "The Modern Management of Some Everyday Obstetrical Problems," N. H. Soble, M. D.

Rochester, N. Y., May 25, 1913.

To the Legal Profession of this County:

Please observe that our society has again prepared a list of experts in insanity for another year. This list is the same as that of last year with one additional name. They are as follows: Edward B. Angell, Eveline P. Ballintine, Edward L. Hanes, Wallace J. Herriman, Eugene H. Howard, P. W. Neefus, Mary C. Nickerson, Sarah G. Pierson, Ezra B. Potter, Alvah C. Remington, Alexander L. Smith, C. A. Van der Beek, Willard H. Veeder and Irving Lee Walker.

Our society is convinced that attempts at the regulation of medical expert testimony may fail for some time to come, and so it has decided to attack the problem in a new way.

The names are first acted upon by our Board of Censors and then elected by the society by ballot after due notice, a two-thirds vote being required to elect. In this way our society feels that it can vouch for these experts. It vouches for no others at present.

You may, of course, use them or not, as you wish. However, the inference is that should you use others no blame can come to our profession because of any medico-legal scandals arising therefrom. Very sincerely, CHARLES W. HENNINGTON,

Secretary Medical Society of the County of Monroe.

